# Optimized fermentation conditions for improved antibody yield in hybridoma cells

**DOI:** 10.1186/1753-6561-7-S6-P74

**Published:** 2013-12-04

**Authors:** Martina Stützle, Alina Moll, René Handrick, Katharina Schindowski

**Affiliations:** 1 Institute of Applied Biotechnology, University of Applied Sciences Biberach, Biberach, 88400, Germany; 2 Medical Faculty, Ulm University, Ulm, 89081, Germany

## Background

Traditionally antibody producing cells like hybridoma cells sank into oblivion since other suspension cell lines have captured the biopharmaceutical production market. However, they are still of particular interest in academic and industrial diagnostic research. Hence, fast and sufficient antibody production is needed as proof of concept, for toxicology and in vivo studies. Although, hybridoma cultivation in fetal bovine serum (FBS) containing animal derived ingredients, like contaminating IgG, is undesirable and leads to difficulties in purification. When reducing the serum to a minimum other key components of the FBS have to be replaced. Therefore, human insulin-like growth factor (IGF) [1] and the surfactant Pluronic F68 were supplemented to improve overall cell performance and to reduce shear stress during shaking respectively employing Design of Experiment (DoE)[2]. Compared to the original basal medium an improvement in cell growth, viability and antibody titer was achieved. These optimized inoculum conditions were used for subsequent bioreactor fermentations. Furthermore, these conditions were used in order to test feeding strategies. For this purpose a fed-batch process with a double bolus feed was simulated in shake flasks with two different glucose feeding strategies - with and without Hyclone Cell Boost 6 (CB6). Finally, the result from shake flasks could be verified and improved antibody yield was achieved in a controlled 2L fed-batch process.

## Material and methods

### DoE approach

DoE (Modde, Umetrics) was used to optimize the cultivation medium by varying the three factors, FBS (1-10%), IGF (10 - 100 μg/L) and Pluronic (0.2 - 1 g/L). The central composite face-centered design was applied to test 24 different medium compositions. Cells were cultivated with a seeding density of 2 × 10^5 ^cells/mL for five days in these media in 40 mL working volume in 125 mL shaker flask. Cell concentration and viability was quantified every day using an image-based cell counter (Cedex XS, Roche) and were defined as response factors for DoE analysis (table 1). Cultures grown with optimized conditions were used as inoculum for subsequent bioreactor fermentations.

**Table 1:** Central composite face-centered result

Exp No	FBS [%]	Pluronic [g/L]	IGF [ug/L)	Viability [%]	Viable cell concentration [cells/mL]
1	-1 (1)	-1 (0.2)	-1 (10)	75.8	736000
2	1 (10)	-1 (0.2)	-1 (10)	79.1	1.468e+006
3	-1 (1)	1 (1)	-1 (10)	66.6	575000
4	1 (10)	1 (1)	-1 (10)	82	1.401e+006
5	-1 (1)	-1 (0.2)	1 (100)	71.6	696000
6	1 (10)	-1 (0.2)	1 (100)	77.9	1.545e+006
7	-1 (1)	1 (1)	1 (100)	59.3	554000
8	1 (10)	1 (1)	1 (100)	78.7	1.319e+006
9	-1 (1)	0 (0.6)	0 (55)	69.1	632000
10	1 (10)	0 (0.6)	0 (55)	79.8	1.455e+006
11	0 (5.5)	-1 (0.2)	0 (55)	82.5	1.461e+006
12	0 (5.5)	1 (1)	0 (55)	78.9	1.442e+006
13	0 (5.5)	0 (0.6)	-1 (10)	79.1	1.326e+006
14	0 (5.5)	0 (0.6)	1 (100)	81.6	1.336e+006
15	0 (5.5)	0 (0.6)	0 (55)	81.8	1.27e+006
16	0 (5.5)	0 (0.6)	0 (55)	80.2	1.194e+006
17	0 (5.5)	0 (0.6)	0 (55)	81.3	1.188e+006

18	0	0.6	55	28.5	13300
19	5.5	0	55	78.4	1.255e+006
20	5.5	0.6	0	81.25	1.2685e+006

21	5.5	0.2	100	83.7	1.533e+006
22	10	0	0	83.6	1.28e+006
23	6	0	0	81.9	1.305e+006
24	1	0	0	57.2	464000

### Feeding strategy

Cells were seeded with 3 × 10^5 ^cells/mL in 35 mL working volume in 125 mL shake flasks in optimized medium (DMEM, 4.5 g/L glucose, 2 mM stable glutamine, 6% FBS, 100 μg/L IGF and 0.2 g/L Pluronic). The 1^st ^triplicate was cultivated without feeding as batch control. The 2^nd ^triplicate was fed with 20 mM glutamine and 20 g/L glucose. The 3^rd ^triplicate was fed with 14 g/L glucose in CB6 (Hyclone, Thermo Scientific) instead of usual glucose feeding in medium. Substrates and metabolites, cell concentration and antibody titer were measured with a chemical analyzer (Konelab, Thermo Scientific), an image-based cell counter (Cedex XS, Roche) and Protein A HPLC (Agilent), respectively.

### Fed-batch with and without Cell Boost 6

Both feeding strategies with and without CB6 were performed again in a 2L bioreactor. The incolumn density was 3 × 10^5 ^cells/mL. The main parameters were kept constant at 1 mM glutamine and at 2 g/L glucose.

## Results and discussion

### DoE approach

A simple DoE approach with the three factors FBS, IGF and Pluronic led to improved hybridoma cultivation conditions. In Table 1 viability and viable cell concentration are depicted from exponential phase for all 24 media on day 3. Additional controls were run to improve the model like zero values for each factor and various FBS concentrations. FBS could be reduced from 10% to 6% by adding 100 μg/L human insulin-like growth factor and 0.2 g/L Pluronic. Compared to the original base medium an improvement in cell growth and viability was achieved.

Three concentration levels for each variable including a maximum (1) a minimum (-1) and a center point (0) were used. Values shown in parenthesis are concentrations. Exp no 15-17 shows the central points for the medium, which were repeated three times. Exp no 18-20 shows the zero controls for each factor. Exp no 21-24 are additional controls for FBS at different concentrations. The concentrations in the yellow and red box are not in brackets. Viability and viable cell concentration were determined as response factors and used for fitting and evaluating the model.

Based on the DoE results, the optimized medium was compared to the original culture conditions with FBS (10%, 6% and 1%) subsequently in 125mL shake flasks in triplicates. Reduction of FBS without supplementation results in decreased viability and cell concentration. The optimized medium, compared to 10% FBS supplementation, showed a significant impact in viable cell concentration and antibody titer by 1.2 fold.

### Feeding strategy

**Figure 1:**
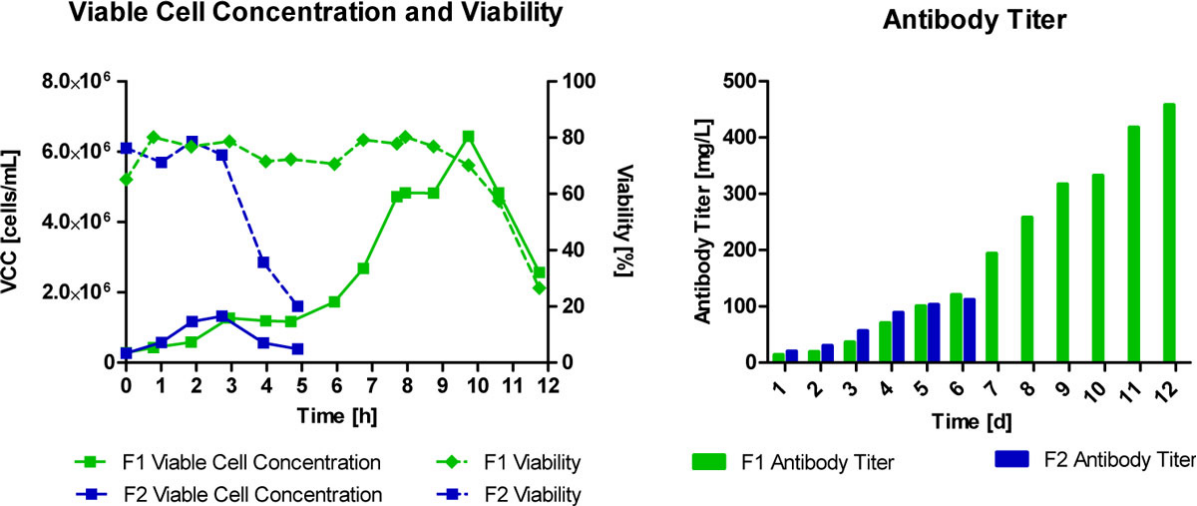
**Fed-batch process with double feed - glutamine in medium and glucose (F1: with CB6; F2: without CB6)**.

After optimizing the inoculum conditions, a fed-batch process was simulated in 125 mL shake flask due to a daily bolus feed with glutamine and glucose. The batch control ended in the death phase at day 3, whereas the fed-batch feed led to 6 day cultivation time. The feeding strategy with CB6 revealed a slightly improved cell growth. This result could be tremendously improved in a controlled 2L bioreactor leading to elongated process time (6 to 12 days), an increased viable cell concentration (from 1.6 × 10^6 ^cells/mL to 6.4 × 10^6 ^cells/mL) and higher antibody titer (450mg/L compared to initial 110mg/L) (Figure 1).

Fed-batch was started with optimized medium (DMEM supplemented with 6% FBS, 100 μg/L IGF and 0.2 g/L Pluronic). Glutamine was hold constant at 1 mM and glucose at 2 g/L. Substrates and metabolites, cell concentration and antibody titer were measured with a chemical analyzer (Konelab, Thermo Scientific), an image-based cell counter (Cedex XS, Roche) and Protein A HPLC (Agilent) respectively each day.

## Conclusion

This data presents DoE as a powerful and efficient time saving tool in process optimization as well as a novel feeding strategy for fed-batch hybridoma process for increased IgG production. By employing DoE, FBS could be decreased from 10% to 6% by 100 μg/L human IGF and 0.2 g/L Pluronic F68. For entirely serum-free hybridoma culture further critical ingredients like transferrin and albumin have to be replaced. However, serum-free media leads to higher production costs and can result in antibody yield reduction. Optimized medium was successfully used for subsequent bioreactor processes starting with a better cell performance. Fed-batch feeding with Hyclone Cell Boost 6 was beneficial for cell growth and antibody production compared to the conventional feed with glucose in medium. Both the optimized medium as well as the Cell Boost 6 feeding strategy led to a prolonged process time and increased antibody titer in the fermentation process.
